# Unique Crystallization of Fullerenes: Fullerene Flowers

**DOI:** 10.1038/srep32205

**Published:** 2016-08-26

**Authors:** Jungah Kim, Chibeom Park, Intek Song, Minkyung Lee, Hyungki Kim, Hee Cheul Choi

**Affiliations:** 1 Center for Artificial Low Dimensional Electronic Systems, Institute for Basic Science (IBS), Pohang 37673, Republic of Korea; 2 Department of Chemistry, Pohang University of Science and Technology (POSTECH), Pohang 37673, Republic of Korea

## Abstract

Solution-phase crystallization of fullerene molecules strongly depends on the types of solvent and their ratios because solvent molecules are easily included in the crystal lattice and distort its structure. The C_70_ (solute)–mesitylene (solvent) system yields crystals with various morphologies and structures, such as cubes, tubes, and imperfect rods. Herein, using C_60_ and C_70_ dissolved in mesitylene, we present a novel way to grow unique flower-shaped crystals with six symmetric petals. The different solubility of C_60_ and C_70_ in mesitylene promotes nucleation of C_70_ with sixfold symmetry in the early stage, which is followed by co-crystallization of both C_60_ and C_70_ molecules, leading to lateral petal growth. Based on the growth mechanism, we obtained more complex fullerene crystals, such as multi-deck flowers and tube-flower complexes, by changing the sequence and parameters of crystallization.

The lattice structure and morphology of a crystal often affect its properties, such as catalytic activity^1^, electrical conductivity^2^,^3^,^4^ and photoluminescence^3^,^4^,^5^,^6^,^7^. Therefore, there has been considerable interest in the development of effective ways to grow crystals with controlled morphology. To obtain such crystals, careful consideration should be given to selecting (1) a proper crystallization method that effectively manipulates the local environment of the growing crystals and (2) a proper set of materials that interact with each other in a controllable, well-understood manner. In terms of the crystallization method, solution-phase crystallization has been regarded as one of the most powerful methods because the use of various solvents can effectively manipulate the local environment of solute molecules. Most examples of solution-phase crystallization occur in a single step; there is no *external* modification or manipulation of conditions once the crystallization begins. Single-step crystallization is straightforward and facile, but it lacks variety of products. Therefore, strategies have been developed for a larger variety of products, such as controlling the ratio of the capping reagent for silver nanoparticles^8^,^9^ or introduction of additional molecules for flower-shaped BSA-Cu_3_(PO_4_)_2_∙3H_2_O crystals^10^. Other examples of solution-phase crystallization take advantage of *external* manipulation; thus, growth occurs in multiple steps. Using multi-step crystallization, one can intentionally design and engineer the crystallization environment to obtain well-defined, unique crystals. For example, flower-shaped BaCO_3_-SiO_2_ structures were obtained by controlling the CO_2_ concentration, pH, and temperature during growth^11^, and other materials, such as metals^12^, oxides^13^, and peptides^14^ were able to adopt this strategy. However, this approach requires in-depth understanding of the target system, and only a few examples have been reported.

To fully exploit the advantages of solution-phase crystallization, one must select a proper set of solute and solvent, of which the mutual interactions are well understood. Highly conjugated fullerenes are one of the most intriguing candidates because they have a wide range of crystallization behaviour depending on the type of the solvent, which is often included in the crystal lattice^6^,^7^,^15^,^16^. Moreover, the addition of poor solvent can further diversify the chemistry because it lowers the solubility of fullerene solutes and alters the local concentration of the solutes. Of various fullerene solution systems, the C_70_ (solute)–mesitylene (solvent) system holds great importance because the crystallization is susceptible to various parameters, such as the solvent ratio and the type of solvent. For example, cube-shaped C_70_ crystals were obtained from mesitylene solution^6^, but the addition of sufficiently poor solvents, such as isopropyl alcohol (IPA) and ethanol, respectively, results in tubes and imperfect rods^7^. The large variety of crystal morphologies implies the presence of multiple crystallographic phases in the C_70_–mesitylene system, and the discovery of a new phase in this system inspired us to design new crystal morphologies.

Herein, we report the growth of fullerene crystals resembling a six-petal flower by co-crystallization of C_60_ and C_70_ molecules (as the solutes) in a mesitylene (as the good solvent)/ethanol (as the poor solvent) system. A series of time-dependent experiments revealed that C_70_ molecules dominantly participate at the initial stage of the crystallization, followed by co-crystallization of C_60_ and C_70_ molecules, which causes lateral growth into *petals*. Based on the crystallization mechanism, we obtained flower-like crystals with plural decks by modulating the morphology of the seed crystals. Further control of the morphology into complex structures was achieved by adopting two-step crystallization.

## Results and Discussion

**Figure 1:**
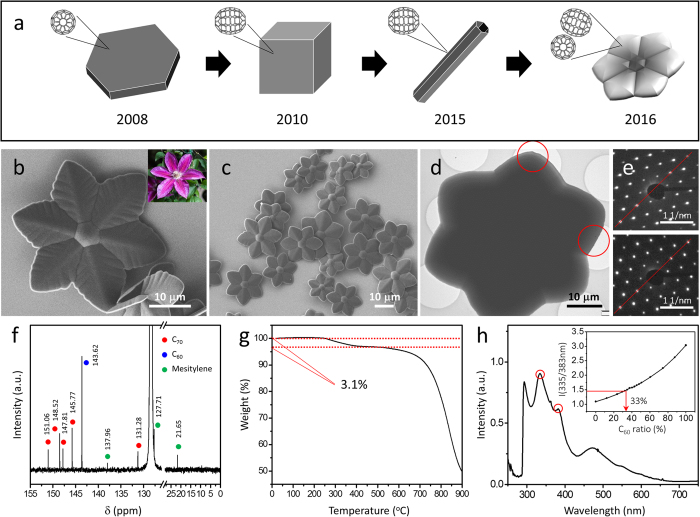
Novel flower-shaped fullerene crystals. (**a**) Research flow of fullerene crystallization in our group. (**b**) SEM images of the flower-shaped crystals (fullerene flower) obtained using C_60_-C_70_-mesitylene/ethanol mixture (Inset: photograph of a clematis flower^18^). (**c**) Low-magnified SEM images of fullerene flowers. The mixing condition does not affect the morphology but does affect the size homogeneity. (**d**) A bright-field TEM image of a typical fullerene flower. (**e**) ED patterns of areas circled in (**d**); the two red lines are exactly the same, *e.g.,* the fullerene flower shows nearly perfect sixfold symmetry. (**f**) ^13^C NMR spectrum of fullerene flowers showing the resonances of C_60_ (red), C_70_ (blue), and mesitylene (green). (**g**) TGA data of fullerene flowers (C_60_:C_70_ = 1:1 in precursor solution) obtained with a temperature increase of 10 °C/min under an N_2_ gas environment. The weight decrease between 250 and 450 °C is due to mesitylene evaporation. (**h**) UV-Vis absorption spectrum of a solution prepared by dissolving fullerene flowers (using a precursor solution of C_60_:C_70_ = 1:1) in mesitylene. The C_60_:C_70_ ratio is estimated at 1:2 according to the intensity ratio of the two peaks with red circles and the inset graph.

For the last decade, we have developed a profound knowledge of the crystallization of fullerene molecules by growing fullerene crystals with various morphologies (Fig. 1a). Our first example was vapor-solid growth of C_60_ hexagonal plates, which is based on a vaporization-condensation-recrystallization (VCR) mechanism^17^. To provide greater variety of the crystals, we adopted solution-phase crystallization. The growth of highly photoluminescent cube crystals made of C_70_ and mesitylene was one of our earliest successes in applying solution-phase crystallization to fullerene molecules^6^. We recently reported the growth of C_70_ hexagonal tubes by changing the amount of poor solvent, which reduces the total amount of mesitylene around C_70_^7^. These examples have shown that solution-phase crystallization (as the method) in the C_70_–mesitylene system (as the target materials) is an excellent candidate for the effective growth of crystals with controlled morphology, as we described above.

Our latest trial was the co-crystallization of C_70_ together with C_60_ in a mesitylene-deficient environment. We first prepared a precursor solution by dissolving both fullerenes in mesitylene and then mixing this solution with ethanol in the ratio of 1:30. As a result of this co-crystallization, unique flower-shaped crystals were obtained (Fig. 1b). These flowers have six petals resemble a natural flower, specifically clematis (Fig. 1b inset)^18^. The yield of fullerene flowers was almost 100%. Although the size of each crystal varied depending on the condition of the supersaturated solution, the morphology was identical (Fig. 1c). Their crystallographic structures were characterized by transmission electron microscopy (TEM) and electron diffraction (ED). Because the crystal is too thick for an electron beam to penetrate, ED patterns were obtained at the thin edges of the petals (Fig. 1d). Electron diffraction patterns taken at a random positions show hexagonal, regular arrays of spots (zone axis of [001], details in Fig. S1). In addition, the orientations of these patterns were almost identical (with a difference of 0.4°), even on different petals of a fullerene flower (Fig. 1e). Therefore, considering the overall morphology of the crystal, we concluded that the flower has nearly perfect sixfold symmetry.

To analyze the chemical composition of the fullerene flowers, we used ^13^C nuclear magnetic resonance (NMR) spectroscopy (Fig. 1f), which revealed one C_60_-related resonance (143.62 ppm), five C_70_-related resonances (131.28, 145.77, 147.81, 148.52, and 151.06 ppm), and three mesitylene-related resonances (21.65, 127.71, and 137.96 ppm), all of which are comparable or identical to previous studies^19^,^20^. Therefore, we concluded that a fullerene flower is composed of C_60_, C_70_ and mesitylene. Co-existence of C_60_ and C_70_ in the fullerene flowers is also indicated by the photoluminescence (PL) spectra (Fig. S2). To analyze whether mesitylene exists in the crystal lattice, which is frequently found in other fullerene crystals^6^,^7^,^15^,^16^, we performed thermogravimetric analysis (TGA) under an N_2_ gas flow (Fig. 1g), which showed a weight loss of 3.1% between 250 and 450 °C. Because the sublimation temperatures of both fullerenes are higher than 450 °C, we attributed this loss to mesitylene. That is, mesitylene is the third component of the crystal, as observed in other solvated crystals^6^,^7^,^16^. To assess the ratio of C_60_ to C_70_, we used UV-Vis absorption at 335 nm and 383 nm, which is related to the C_60_/[C_60_ + C_70_] ratio (Fig. 1h inset, Fig. S3). By interpolation, we estimated that the C_60_:C_70_ ratio was 1:2 (Fig. 1h). Combined with the TGA data, we concluded that the molar ratio of C_60_:C_70_:mesitylene in the fullerene flowers is 1:2:2.

**Figure 2:**
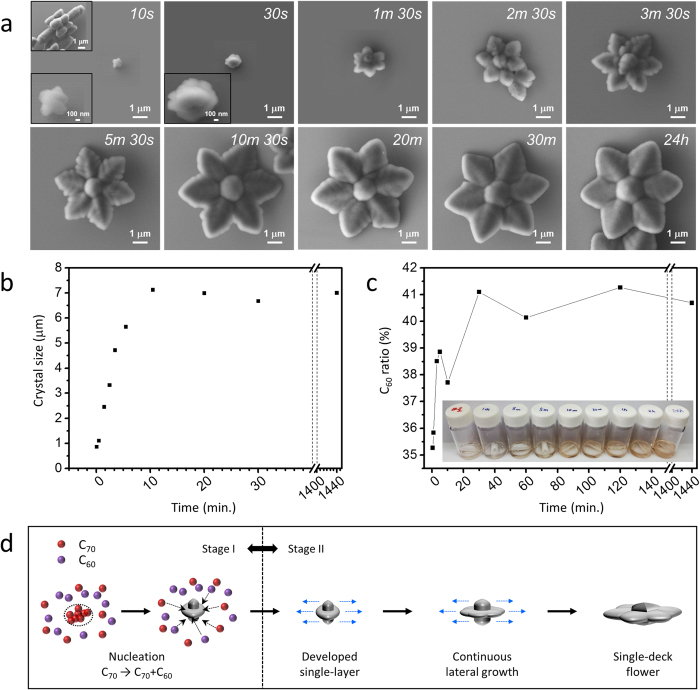
Crystallization mechanism of fullerene flowers. (**a**) A series of SEM images of fullerene crystals obtained at different growth times after sonication. The flower-like shape develops within 2 min, and the lateral growth stops at 10 min. The C_70_ crystal resembles the initial crystal (10 s image) shown in the top left inset. (**b**) Time-dependent crystal size evolution estimated by averaging the diameters of three random fullerene flowers. (**c**) Time evolution of the C_60_ ratio estimated by UV-Vis absorption. The graph could be obtained using the relation between C_60_/[C_60_ + C_70_] and the intensity ratio of 335 nm/383 nm (details in Fig. S3). The increase of the C_60_/[C_60_ + C_70_] ratio may be due to dominant C_70_ participation in the early stage of crystallization. (**d**) Schematic illustration of the crystallization mechanism of a fullerene flower.

Time-dependent morphologies observed by scanning electron microscopy (SEM) showed that the flower-like shape developed within 2 minutes after 20-second sonication, and that crystals did not grow after 10 minutes (Fig. 2a,b). To carefully examine the origin of such flower-like growth, we focused on the first 2 minutes after sonication. The crystal seed obtained immediately after sonication morphologically resembled the pure C_70_ crystals (the top left inset in the first image) obtained in the same system (mesitylene/ethanol)^7^. These seed crystals then laterally grew to become flower-like crystals (Fig. 2a). In addition, UV-Vis absorption spectroscopy revealed that the proportion of C_60_ in the crystals gradually increased as the crystallization proceeded (Fig. 2c). Therefore, we concluded that at the initial nucleation stage, the seed is essentially a pure C_70_ crystal, and during the following growth stage, lateral growth of the crystal occurs by attachment of C_60_ onto the lateral ends of seeds (Fig. 2d). It was practically impossible to obtain fullerene crystals with a C_60_/[C_60_ + C_70_] ratio lower than ~35% because the minimum pore size of available filter papers is limited (0.1 μm), and some of the crystals may have been nucleated during sonication. Nonetheless, such incomplete observations support our two-stage crystallization mechanism.

We assume that the solubility difference of C_60_ and C_70_ in mesitylene/ethanol system^21^ explains the origin of the unique two-stage growth. Immediately after injection of the C_60_-C_70_-mesitylene solution into ethanol under strong sonication, locally excessive ethanol breaks the mesitylene solvent shell around the fullerenes. Because the solubility of C_70_ in ethanol is lower than C_60_^21^, C_70_ molecules are primarily precipitated out of the shell to form C_70_-dominant imperfect rods (*i.e.,* the nucleation stage). After complete mixing of both solvents, the instability of the solvated C_70_ is comparable to that of C_60_; thus, they co-precipitate into plates (details in Fig. S4). In contrast, when a slower crystallization method, such as liquid-liquid interfacial precipitation (LLIP), is used, the tendency of these solutes to crystallize is not significantly large; thus. the resulting crystals have uniform morphology with a homogeneous composition (Fig. S5)^22^,^23^. This two-stage crystallization simulates complex multi-step crystallization without any external modulation of the growth conditions.

However, the origin of the unique, complex morphology of our crystals is not fully explained by the two-stage mechanism described above because the presence of multiple stages does not guarantee the complex morphology of the resulting crystals. For example, C_60_ and C_70_ in a mesitylene/isopropyl alcohol (IPA) system only yield hexagonal tubes, regardless of the composition of the solutes [0%, 50%, and 100% of C_60_/(C_60_ + C_70_)] (Fig. S6). That is, if both nucleation and growth stages prefer the identical morphology, which is a hexagonal tube in this case, the morphology of growing crystals will not change or *evolve* as crystallization proceeds. The key requirement for the complexity, therefore, would be the difference of the morphology that each stage prefers. Indeed, the morphology of single crystals obtained from pure C_70_ in the mesitylene/ethanol system, which would represent the preferred morphology at the nucleation stage, is an imperfect rod, but that of the mixture of C_60_ and C_70_, reflecting the growth stage, is a plate (Fig. S5). In accordance with the time-dependent morphology study, a fullerene flower can be effectively reduced to a combination of a hexagonal center and six plates. Because six bumps on the imperfect rods are unstable, newly precipitating molecules attach to them to grow into plates and ultimately produce a flower.

The mechanism above demonstrates the active role of the poor solvent, ethanol in our case, throughout the whole crystallization in this binary solute (where solute A is less soluble in the poor solvent than is solute B) – binary solvent (good and poor solvent) system. Upon injection of the poor solvent into the solute-good solvent solution, the local excess of the poor solvent breaks the solvent shell around the solutes and promotes precipitation of A. The resulting crystals resemble the crystals that are obtained without B (this is the nucleation stage). Upon complete mixing of both solvents, the preferred morphology of the growing crystals resembles crystals obtained from slow crystallization (this is the growth stage). That is, both quick and slow crystallization eventually yield similar crystal shapes, but the use of a poor solvent in the quick crystallization *holds* it for a while and provides chemical environment such that B becomes chemically inert at the nucleation stage. This role of the poor solvent has been veiled so far because the first stage is very quick and the overall morphology does not evolve. Ethanol, however, can differentiate the morphologies of each stage and provides the opportunity to discover it. Therefore, we concluded that the role of the poor solvent is much more active than previously expected; thus, one should consider the poor solvent in controlling the crystallization and in proposing a valid crystallization mechanism.

**Figure 3:**
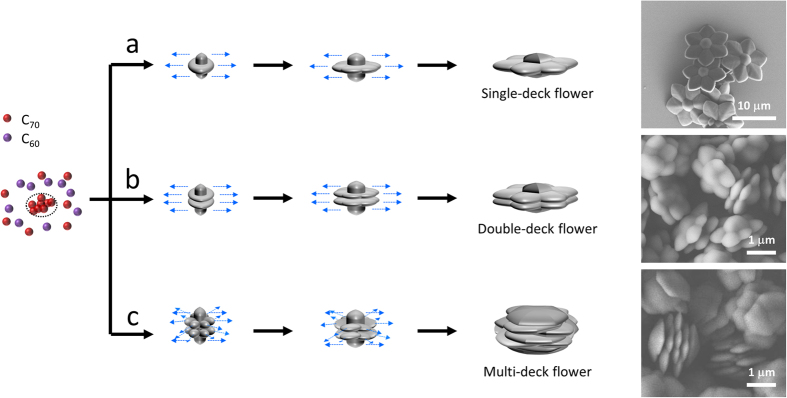
Controlling the number of decks of fullerene flowers. Crystallization begins by the nucleation of C_70_ molecules followed by lateral growth with both C_60_ and C_70_. Because single-deck formation originates from the single lateral layer of a C_70_ crystal, it is expected that the deck of a fullerene flower can be controlled by changing the morphology of the C_70_ crystals formed at this early stage. (**a**) When 0.1 mM of C_70_-mesitylene is used, one-layered nuclei are developed and become single-deck fullerene flowers. (**b**) Double-deck fullerene flowers are formed when a higher concentration of C_70_-mesitylene (0.2 mM) is used because the nuclei have a double-layered character. (**c**) The lateral growth after the full formation of C_70_ bumpy rods can develop multi-deck fullerene flowers due to the staggered configuration of the twelve bumps.

Based on the proposed mechanism, we assumed that further modification of the experimental processes may yield *modified* fullerene flowers, and we obtained fullerene flowers with an altered shape. First, we increased the number of the decks of fullerene flowers by decreasing the ratio of C_60_/[C_60_ + C_70_] in the precursor solution (Fig. 3). As we observed in the early stage of crystallization, the lateral sides of the seeds act as nucleation sites for lateral growth (see above). Here, we found a clue to add an extra deck of petals from our previously reported pure C_70_ crystals in the same system^7^, which show remarkable resemblance with our flower seeds. A low concentration of C_70_ (≤0.1 mM) results in imperfect rods, but a higher concentration (>0.1 mM) forms an extra set of six bumps (Fig. S7). Based on this assumption, we decreased the ratio of C_60_ with respect to C_70_, and we successfully obtained double-deck flowers (Fig. 3b). To analyze the effect of the amount of C_70_ on the nucleation process, the C_60_/[C_60_ + C_70_] ratio of the precursor solution was varied from 0% to 100%. Then, the morphology of the obtained crystal changed as follows: a bumpy rod (0%), a single-deck flower (≤50%), a double-deck flower (>50%), and a hexagonal tube (100%) (Fig. S8). A higher ratio results in larger crystals because it reduces the number of seeds by decreasing the supersaturation level^6^,^7^,^24^,^25^.

To further increase the number of decks, we separated the nucleation and growth stages into distinct experimental steps^13^,^26^. Such deliberate separation of crystallization stages allows lateral growth onto completely developed pure C_70_ bumpy rods (Fig. S7). The first step was making C_70_ bumpy rods by mixing C_70_-mesitylene/ethanol at 1:30. The rods were filtered out of the solution and re-dispersed in 2 mL of ethanol. We placed the C_70_ bumpy rods into filtered C_60_-C_70_-mesitylene/ethanol as the second step, and multi-deck flowers were obtained (Fig. 3c). Syringe filters with a pore diameter of 0.02 μm can effectively remove the seeds generated in the C_60_-C_70_-mesitylene/ethanol; thus, the filtered solution, which is supersaturated, can directly participate in the lateral growth process. The staggered geometry of the flower decks can be attributed to the staggered geometry of the lateral bumps.

**Figure 4:**
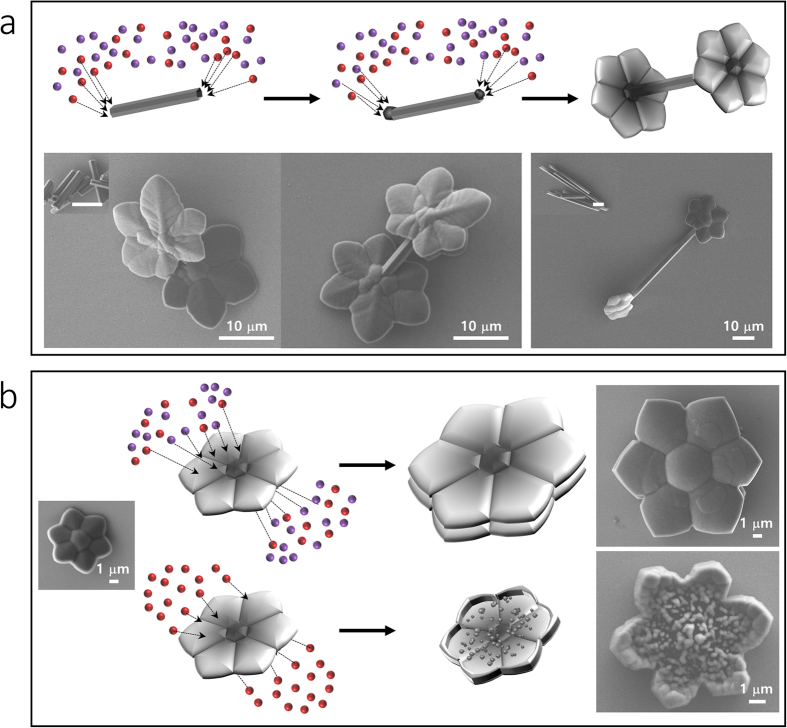
Further crystallization controls with fullerene flowers. (**a**) Schematic illustration of fullerene flower crystallization on the tips of a C_60_ hexagonal tube and SEM images of the obtained results. When C_60_ hexagonal tubes are used as crystal nuclei, fullerene flowers are crystallized at their tips, which have relatively high surface energy. Two C_60_ hexagonal tubes with different lengths were used for comparison. (**b**) A further increase in size is achieved by attachment of both C_60_ and C_70_ to the pre-crystallized fullerene flowers, which also yields a double-deck shape because the crystallization process occurs at each side of the fullerene flowers. In addition, due to the high surface energy of the edges of the flowers, C_70_ molecules dominantly attach to the edges, resulting in edge-grown flowers.

Last, we obtained flower-containing heterostructures, such as a flowers with a stem, by changing the crystal seeds (Fig. 4a, scheme). C_60_ hexagonal tubes, whose tips have high surface energy^26^,^27^,^28^, were used as the crystallization seed and ultimately the stem (Fig. 4a insets). Filtered C_60_-C_70_-mesitylene/ethanol was added to these tubes, and a pair of fullerene flowers “bloomed” at both tips (Fig. 4a). Another trial was performed using the fullerene flower itself as the seed for modification. When the single-deck fullerene flowers were mixed with filtered C_60_-C_70_-mesitylene/IPA, double-deck flowers with increased size were obtained (Fig. 4b, top). However, when mixed with C_70_-mesitylene/ethanol instead, clusters of C_70_ were attached dominantly on the edges of the fullerene flowers (Fig. 4b, bottom). The fullerene flowers did not undergo any morphological changes when C_60_-mesitylene/ethanol solution was used. These results suggest that the additional growth of C_70_ on the fullerene flower may be due to the similarity between the structures of the fullerene flower and the C_70_ crystals in mesitylene/ethanol.

## Conclusion

We made highly symmetrical flower-shaped fullerene crystals starting from mesitylene/ethanol solutions of mixed C_60_ and C_70_. The crystals consisted of both C_60_ and C_70_, with solvation by mesitylene. The main factor behind the unique crystal growth was the solubility difference between C_60_ and C_70_, which led to the two-stage growth: first C_70_ formed the center of the crystal, and then C_60_-C_70_ co-crystallized to form petals. The poor solvent, ethanol in our case, guided the crystallization mechanism to yield complex flower crystals, which is surprising because of the role of the poor solvent in affecting the crystallization pathway. By manipulating the morphology of the crystals on which the petal formation occurs, various fullerene flowers could be obtained: single-deck, double-deck, and multi-deck fullerene flowers. In addition, we synthesized more complex crystals by adopting a two-step growth process. The high surface energy of the tips of C_60_ hexagonal tubes resulted in a pair of fullerene flowers grown on each tip of a stem. The use of different types of solutions can effectively alter pre-grown fullerene flowers into larger fullerene flowers or edge-grown fullerene flowers. We believe that our findings provide insights into the solution-phase growth of molecular crystals and illuminate new aspects of crystallization chemistry.

## Materials and Methods

### Fullerene flowers

C_60_ (99.95%, MTR Ltd, Cleveland, Ohio, US) and C_70_ fullerene powder (99.0%, MTR Ltd., Cleveland, Ohio, US) were used as received. Each fullerene was dissolved in mesitylene (98%, Alfa Aesar, Heysham, Lancashire, UK) at room temperature under ultrasonication for 2 h; then, the solutions were filtered (syringe filter, pore: 0.02 μm, Whatman, Maidstone, Kent, UK) to remove invisible residue and undissolved fullerenes. The concentrations of the solutions were measured using UV-Vis absorption spectra and were then diluted with mesitylene to concentrations of 0.1–0.2 mM. C_60_-mesitylene and C_70_-mesitylene solutions with the same molarity were mixed at volume ratios of 1:1 and were then mixed with ethanol (99.9%, Fisher Scientific, Pittsburgh, Pennsylvania, US) in a 1:30 ratio (fullerene solution:ethanol v/v) under ultrasonication for 20 s. The mixed solutions were kept at room temperature for 24 h, and precipitated fullerene flowers were pipetted into appropriate substrates for further characterization.

### Crystallization mechanism

First, scanning electron microscope (SEM, JEOL, Tokyo, Japan) images of the fullerene crystals were captured at ten different times during the crystallization into fullerene flowers. Sample preparation on the silicon substrate was as follows. From 10 s to 10.5 min after the initial 20 s sonication, 2 mL of the mixed solutions was filtered (pore size: 0.01 μm), and crystals in the filtrate were transferred to a silicon substrate. Crystals obtained after 10.5 min, which were observed as precipitate at the bottom of the vial, were directly transferred to the silicon substrate.

Second, UV-Vis absorption analysis (Agilent 8453, Agilent, Santa Clara, California, US) was performed to confirm the composition ratio of C_60_ and C_70_ in the crystals. We prepared nine identical precursor solutions, then filtered them at nine different times after 20 s sonication. Crystals on the filter were fully dissolved in mesitylene and were analyzed using the intensity ratio of 335 nm/383 nm from the UV-Vis absorption spectra.

Third, to confirm the effect of the crystallization method, LLIP was performed using 0.17 mM C_60_-C_70_-mesitylene and ethanol. We are slowly added 0.5 mL of C_60_-C_70_-mesitylene through the vial wall, which contained 15 mL of ethanol. Then, the solution was kept for 24 h at room temperature.

Fourth, to confirm the effect of the poor solvent, C_60_-C_70_-mesitylene (0.17 mM, 0.5 mL) and IPA (15 mL) were mixed by 20 s sonication.

### Crystallization of single-deck, double-deck, and multi-deck fullerene flowers

Single-deck and double-deck fullerene flowers: C_60_/[C_60_ + C_70_] ratio-dependent crystallization was performed using six precursor solutions: 0%, 20%, 33%, 50%, 67%, and 100%. Each solution was prepared by mixing 0.17 mM C_60_-mesitylene and C_70_-mesitylene at ratios of 0:1, 1:4, 1:2, 1:1, 2:1, and 1:0 (v/v).

Multi-deck fullerene flowers: In contrast to the crystallization of single-deck and double-deck flowers, multi-deck fullerene flowers were obtained using a two-step crystallization. As the first step, C_70_ bumpy rods were prepared by mixing C_70_-mesitylene (0.2 mM) and ethanol in a 1:30 (v/v) ratio. They were re-dispersed in 2 mL of ethanol, and 20 μL was transferred to a new vial. In the second step, a second precursor solution, which was used for making fullerene flowers (0.17 mM, C_70_:C_60_ = 1:1, fullerene solution:ethanol = 1:30), was filtered (vacuum filter, pore size: 0.1 μm) within 3 minutes after 20 s sonication. The filtered solution was directly dropped into a vial containing C_70_ bumpy rods dispersed in 20 μL of ethanol.

### Fullerene flowers connected to C_60_ hexagonal tubes

As the first step, C_60_ hexagonal tubes were prepared by mixing C_60_-mesitylene (0.3, 0.2, and 0.1 mM) and ethanol in a 1:15 (v/v) ratio. C_60_ hexagonal tubes were re-dispersed in 2 mL of ethanol, and 20 μL was transferred to a new vial. As the second step, the solution mixture of fullerene flowers (0.17 mM, C_70_:C_60_ = 1:1, fullerene solution:ethanol = 1:30) was filtered (syringe filter, pore size: 0.02 μm) and directly dropped into a vial that contained C_60_ hexagonal tubes dispersed in 20 μL of ethanol.

### Other crystals grown from fullerene flowers

As the first step, we obtained single-deck fullerene flowers from a mixture of 0.5 mL of fullerene solution (0.17 mM, C_70_:C_60_ = 1:1) and 15 mL of ethanol after 20 s of sonication. The obtained crystals were filtered and re-dispersed in 2 mL of ethanol for further growth; then, 50 μL was transferred into a new vial. Fullerene solution (0.17 mM C_60_-C_70_-mesitylene and 0.1 mM C_70_-mesitylene) of 0.5 mL was mixed with 15 mL of poor solvent (IPA and ethanol) with sonication for 20 s, and the solution was filtered within 3 min. The filtered solution (syringe filter, pore size: 0.02 mm) was directly dropped into a vial that contained fullerene crystals, which was manually shaken for 20 s.

### Characterization

The crystal morphologies were confirmed by SEM (JEOL, JSM-7410F). The crystallinity was examined using TEM (EM 912 omega, Carl Zeiss, Oberkochen, Baden-Württemberg, Germany), SAED, and XRD [(5D beamline at Pohang Accelerator Laboratory, λ = 1.2395 Å)]. For easy comparison with previous reports, the X-ray wavelength was scaled to CuKα (λ = 1.54057 Å) radiation. ^13^C-NMR analysis was used to investigate the fullerene flowers’ composition. ^13^C NMR is appropriate for this study because the resonance patterns of C_60_, C_70_, mesitylene, and ethanol are distinct. Solutions for ^13^C NMR analysis were prepared by dissolving filtered fullerene flowers in C_6_D_6_ solvent. UV-Vis absorption spectroscopy was used to measure the concentration of fullerene solution and the ratio of C_60_ and C_70_. TGA analysis was performed with a temperature increase of 10 °C/min under an N_2_ gas environment to confirm the mesitylene information in the crystals.

## Additional Information

**How to cite this article**: Kim, J. *et al*. Unique Crystallization of Fullerenes: Fullerene Flowers. *Sci. Rep.* **6**, 32205; doi: 10.1038/srep32205 (2016).

## Supplementary Material

Supplementary Information
