# Supplementary material for: Unique Crystallization of Fullerenes: Fullerene Flowers
Source: Sci Rep. 2016 Aug 26;6:32205. doi: 10.1038/srep32205 (PMC4999865; doi:10.1038/srep32205)
Supplement: Supplementary Information [file srep32205-s1.pdf]

Supplementary Information

Unique Crystallization of Fullerenes: Fullerene Flower

Jungah Kim[1,2], Chibeom Park[1], Intek Song[1,2], Minkyung Lee [1,2], Hyungki Kim[1,2], and Hee Cheul Choi[1,2]*

[1]Center for Artificial Low Dimensional Electronic System, Institute for Basic Science (IBS), Pohang 37673, Republic of Korea
[2]Department of Chemistry, Pohang University of Science and Technology (POSTECH), Pohang 37673, Republic of Korea

E-mail: choihc@postech.edu

Supporting Figures

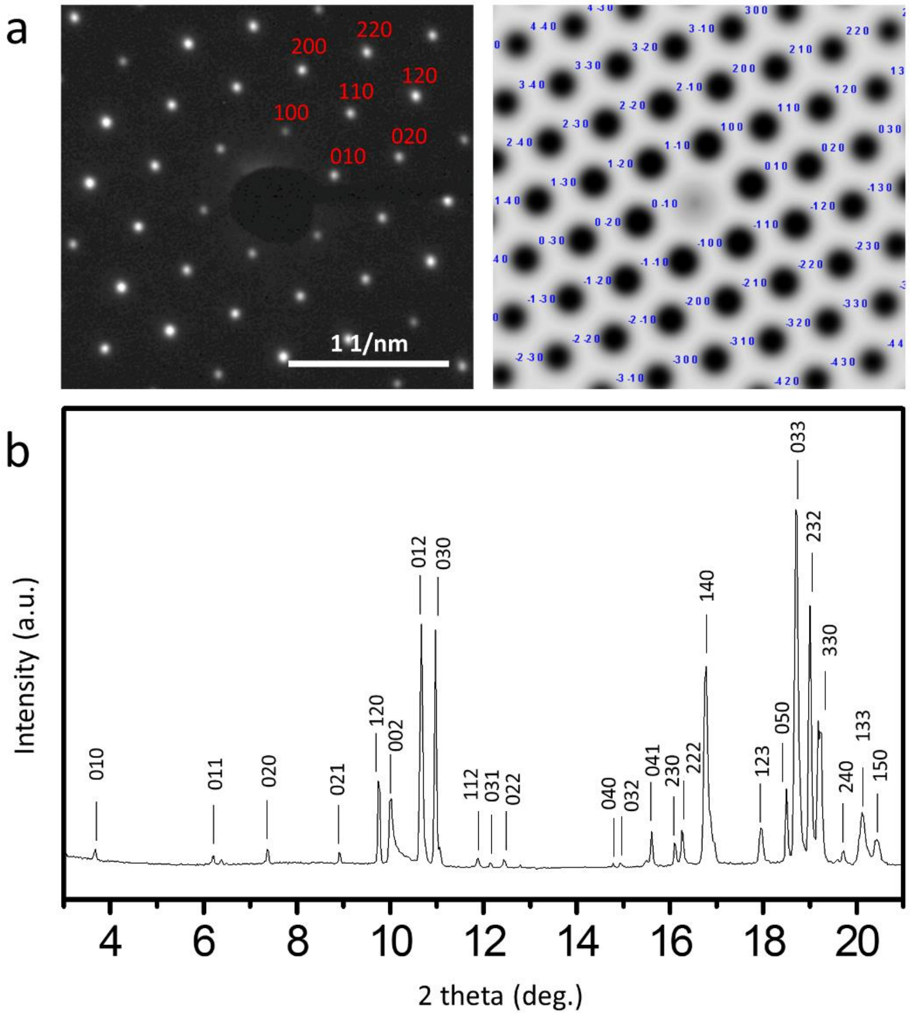

Figure S1. Crystallographic analysis of fullerene flowers. (**a**) The selected area electron

diffraction (SAED) pattern of a fullerene flower (left) and the simulated diffraction pattern based on the lattice constants with the zone axis of [001] (right). CrystalMaker software package (CrystalMaker 2.1.4 and SingleCrystal 1.1.3) was used to simulate the diffraction pattern. (**b**) The powder X-ray diffraction (XRD) pattern of fullerene flowers with crystallographic indices. To determine the index of each peak, the plane-spacing equation along with Bragg's law was used[1]. All peaks were indexed well when the crystal structure was assumed hexagonal. The determined lattice constants are a = 27.71 Å, c = 17.62 Å (a/c = 1.57), which are different from those of other mesitylene-solvated fullerene crystals.

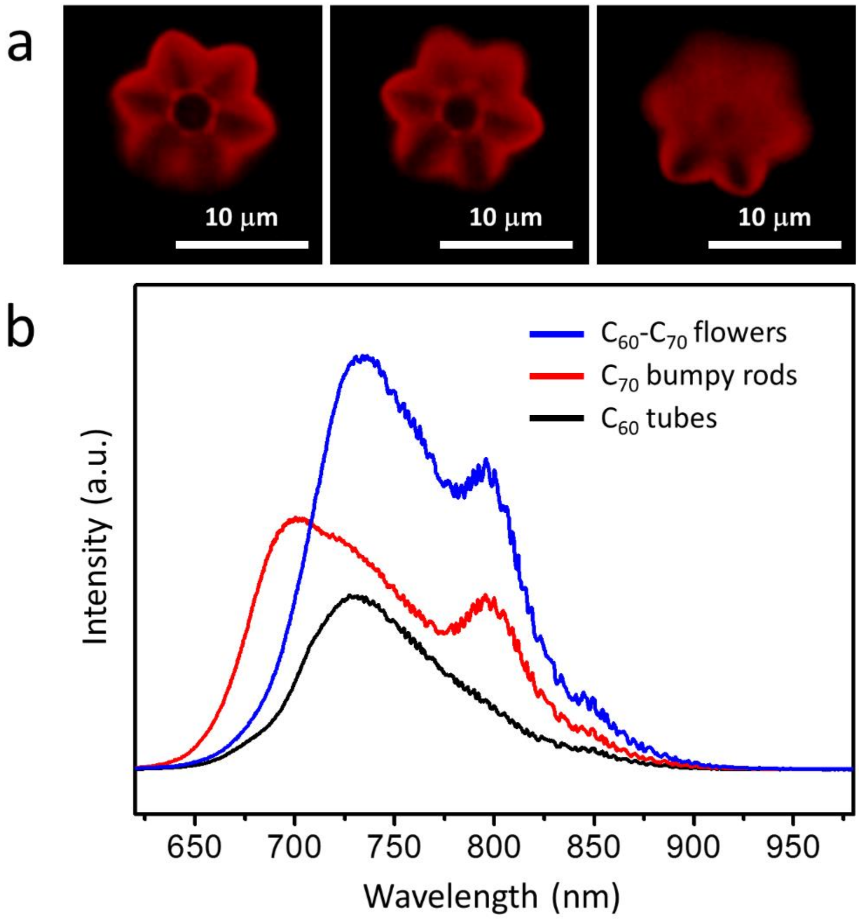

Figure S2. Photoluminescence property of fullerene flowers. (**a**) Photoluminescence (PL) images of a fullerene flower. Because of the tilt of the lying flower and the shall depth of field of the microscope, we took three different PL images by adjusting the object distance. The higher brightness of the edges may be attributed to the waveguiding phenomenon. (**b**) PL spectra of our fullerene flowers and other fullerene crystals. The similarity of the flower spectrum to that of the C_{70} bumpy rods implies that C_{70} is the major component of fullerene flowers. In addition, the red shift with respect to the C_{70} bumpy rods may be due to the presence of C_{60} in the crystal lattice of fullerene flowers.

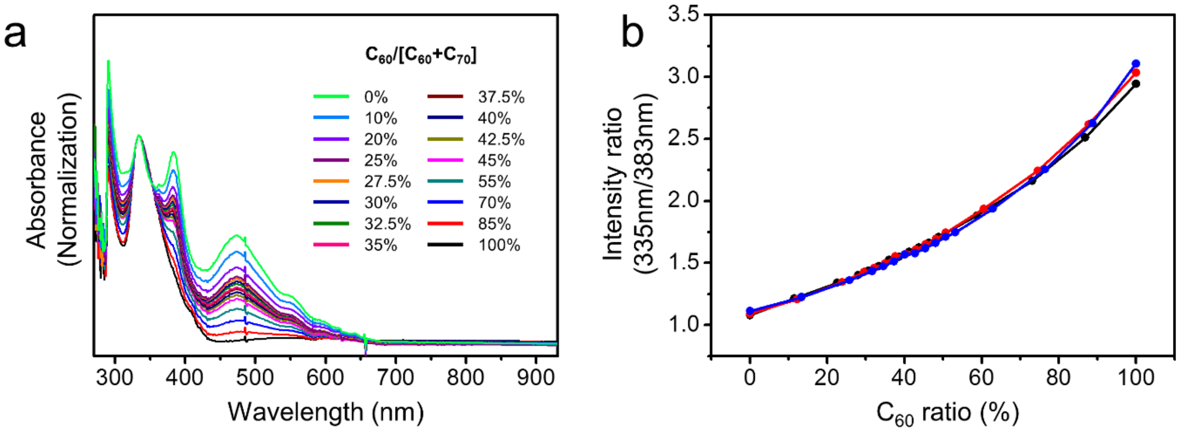

Figure S3. The relation between the UV-Vis absorption and the fullerene ratio. (**a**) A series of UV-Vis absorption spectra of solutions having different ratios of C_{60}/[C_{60}+C_{70}]. All graphs were normalized to the peak intensity at 335 nm. (**b**) The intensity ratio (λ = 335 nm / λ = 383 nm) with respect to C_{60}/[C_{60}+C_{70}] ratio. Note that we prepared three different sets of samples because it is difficult to prepare the identical solutions.

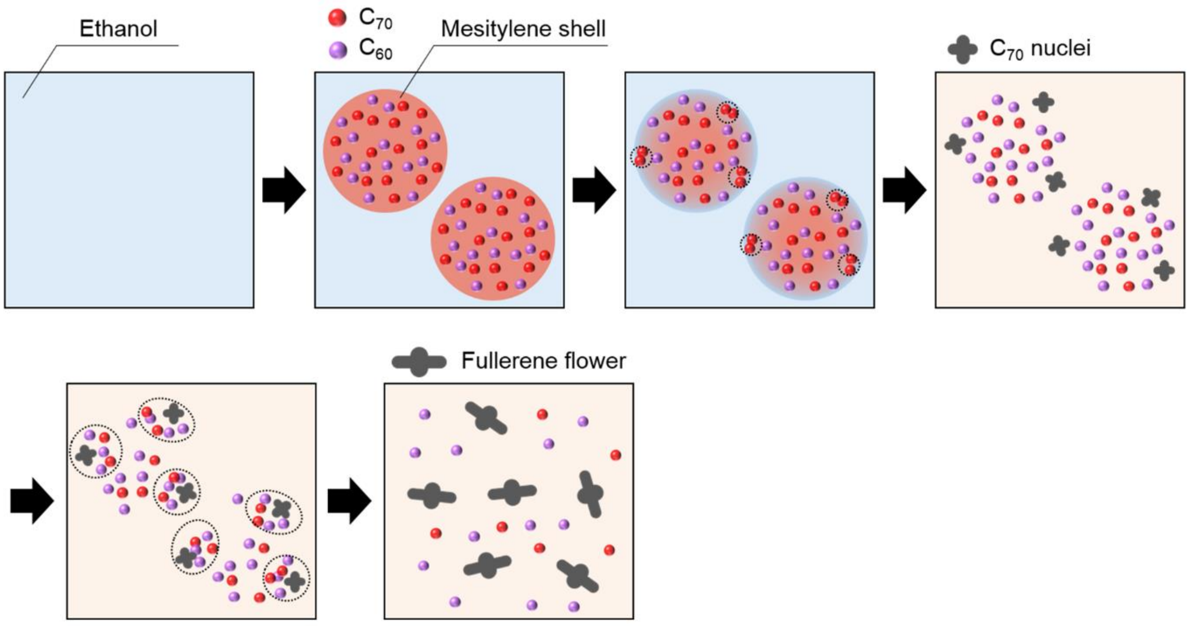

Figure S4. Schematic illustration of the two-stage growth using the solvent shell concept. When C_{60}-C_{70}-mesitylene solution is injected into ethanol, ethanol diffuses into the solvent shell and breaks it. Since the solubility of C_{70} is lowered upon mixing with the poor solvent, ethanol, it now precipitates and coagulates into imperfect rods. After ethanol and mesitylene mix thoroughly, both C_{60} and C_{70} precipitate into petals of the flowers.

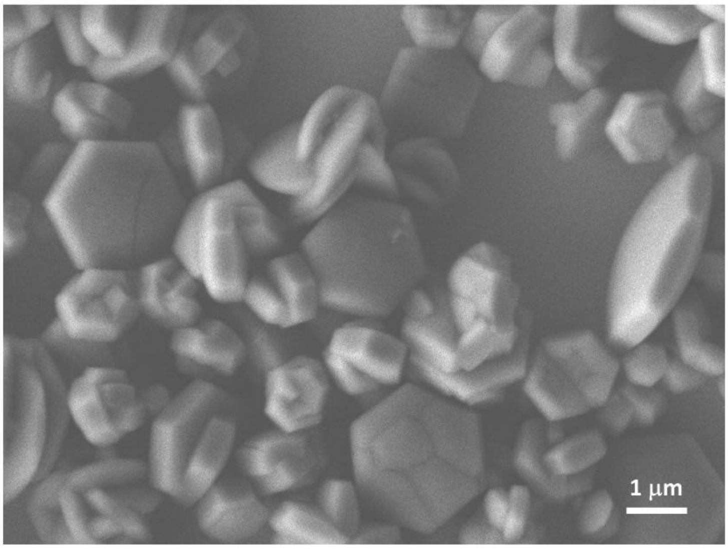

Figure S5. Fullerene crystals obtained using LLIP method. We obtained plate-like crystals using LLIP method for mixing C_{60}-C_{70}-mesitylene and ethanol, and it is due to homogeneous composition of C_{60} and C_{70} in the crystals.

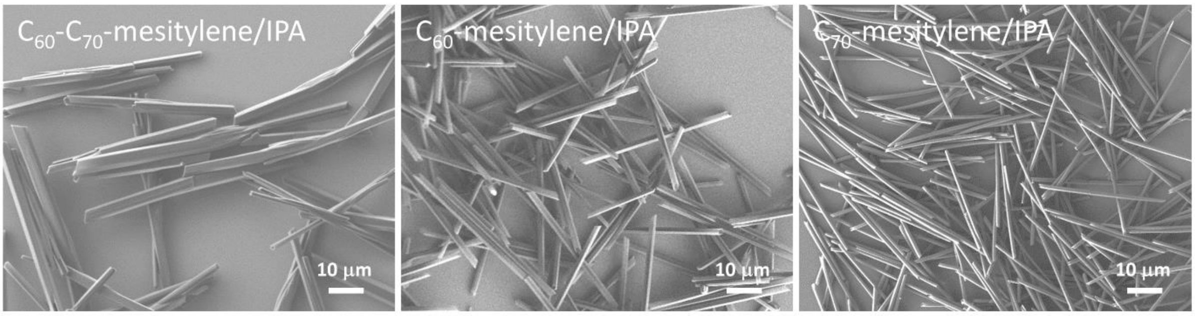

Figure S6. Fullerene crystals obtained from mesitylene/IPA system. Crystals obtained using C_{60}-C_{70}-mesitylene/IPA and one-type of fullerene-mesitylene/IPA. In contrast to the result using ethanol, all of them have similar shape (hexagonal tube).

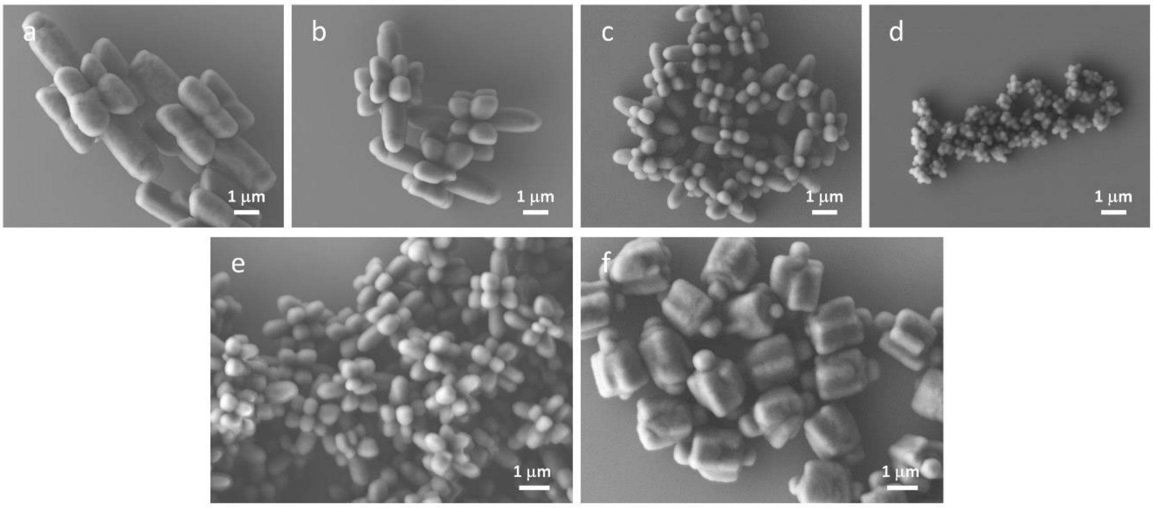

Figure S7. SEM images of pure C_{70} crystals. C_{70} crystal from C_{70}-mesitylene/ethanol at 1:30 volume ratio. Each image obtained using different concentration of C_{70}-mesitylene solution: (**a**) 0.0095 mM, (**b**) 0.12 mM, (**c**) 0.14 mM, and (**d**) 0.17 mM. Another set of six bumps were observed when using higher concentration of C_{70}-mesitylene (> 0.1 mM). Crystals having more bumps is because of sufficient growth, which can be confirmed by additional experiment. **(e) and (f)**. (**e**) shows C_{70} bumpy rods used as seed, and (**f**) shows C_{70} crystals further grown from crystals in (**e**).

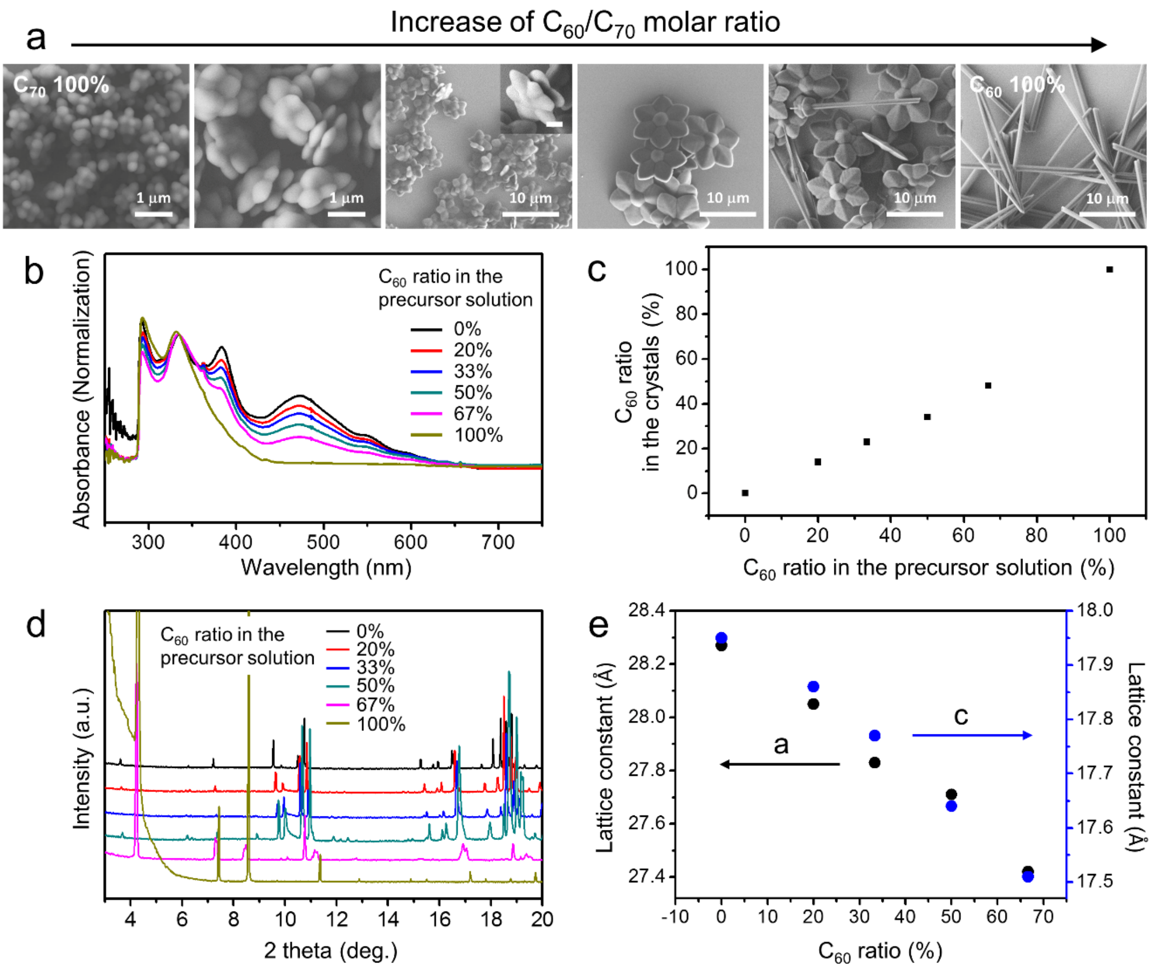

Figure S8. C60 ratio in precursor solution dependent crystallization result and analysis. (**a**) SEM images of fullerene crystals obtained at different ratios of C_{60}/[C_{60}+C_{70}] (left to right: 0%, 20%, 33%, 50%, 67%, and 100%) with enlarged image (inset, scale bar 10 µm) of crystals obtained using C_{60} ratio of 33%. (**b**) UV-Vis absorption spectra of solutions made by re-dissolving fullerene crystals in mesitylene. Each fullerene crystal was crystallized in the precursor solutions containing C_{60} of 0%, 20%, 33%, 50%, 67%, and 100%. (**c**) C_{60} ratio in crystals vs. C_{60} ratio in precursor solution. (**d**) XRD data of crystals shown in A. (**e**) Lattice constants (a and c) of fullerene crystals obtained using solutions containing C_{60} of 0%, 20%, 33%, 50%, and 67%. Lattice constants (a and c) were decreased as C_{60} ratio in the precursor solution increased, but all crystals have the same aspect ratio (a/c = 1.57).

Reference

1. Cullity, B. D. *Element of X-ray Diffraction* (Addison-Wesley Inc., Manila, ed. 2, 1978)
